# Flutamide-induced alterations in transcriptional profiling of neonatal porcine ovaries

**DOI:** 10.1186/s40104-019-0340-y

**Published:** 2019-04-03

**Authors:** Katarzyna Knapczyk-Stwora, Anna Nynca, Renata E. Ciereszko, Lukasz Paukszto, Jan P. Jastrzebski, Elzbieta Czaja, Patrycja Witek, Marek Koziorowski, Maria Slomczynska

**Affiliations:** 1 Department of Endocrinology, Institute of Zoology and Biomedical Research, Jagiellonian University in Krakow, Gronostajowa 9 Street, 30-387 Krakow, Poland; 2 Laboratory of Molecular Diagnostics, Faculty of Biology and Biotechnology, University of Warmia and Mazury in Olsztyn, Olsztyn, Poland; 3 Department of Animal Anatomy and Physiology, Faculty of Biology and Biotechnology, University of Warmia and Mazury in Olsztyn, Olsztyn, Poland; 4 Department of Plant Physiology, Genetics and Biotechnology, Faculty of Biology and Biotechnology, University of Warmia and Mazury in Olsztyn, Olsztyn, Poland; 5 Department of Physiology and Reproduction of Animals, University of Rzeszow, Rzeszow, Poland

**Keywords:** Flutamide, LncRNA, Ovary, Pig, RNA-Seq, Transcriptome

## Abstract

**Background:**

Androgens are involved in the regulation of ovarian development during fetal/neonatal life. Environmental chemicals displaying anti-androgenic activities may affect multiple signal transduction pathways by blocking endogenous androgen action. The aim of the current study was to examine effects of the anti-androgen flutamide on the expression of coding transcripts and long non-coding RNAs (lncRNAs) in neonatal porcine ovaries. By employing RNA-Seq technology we aimed to extend our understanding of the role of androgens in neonatal folliculogenesis and examine the impact of the anti-androgen flutamide on ovarian function.

**Method:**

Piglets were subcutaneously injected with flutamide (50 mg/kg BW) or corn oil (controls) between postnatal days 1 and 10 (*n* = 3/group). Ovaries were excised from the 11-day-old piglets and total cellular RNAs were isolated and sequenced.

**Results:**

Flutamide-treated piglet ovaries showed 280 differentially expressed genes (DEGs; *P*-adjusted < 0.05 and log_2_ fold change ≥1.0) and 98 differentially expressed lncRNAs (DELs; *P*-adjusted < 0.05 and log_2_FC ≥ 1.0). The DEGs were assigned to GO term, covering biological processes, molecular functions and cellular components, which linked the DEGs to functions associated with cellular transport, cell divisions and cytoskeleton. In addition, STRING software demonstrated strongest interactions between genes related to cell proliferation. Correlations between DEGs and DELs were also found, revealing that a majority of the genes targeted by the flutamide-affected lncRNAs were associated with intracellular transport and cell division.

**Conclusions:**

Our results suggest that neonatal exposure of pigs to flutamide alters the expression of genes involved in ovarian cell proliferation, ovarian steroidogenesis and oocyte fertilization, which in turn may affect female reproduction in adult life.

**Electronic supplementary material:**

The online version of this article (10.1186/s40104-019-0340-y) contains supplementary material, which is available to authorized users.

## Background

Many environmental chemical compounds may interfere with the human and animal endocrine system causing serious reproductive problems. These compounds, frequently referred to as endocrine-active chemicals (EACs), display estrogenic/antiestrogenic and/or androgenic/antiandrogenic activities, and as such may affect multiply signal transduction pathways by mimicking or blocking the action of endogenous steroids [1]. It is evident that animals are especially susceptible to EACs during the fetal or neonatal period when even a low-dose exposure may produce adverse effects due to the immature reproductive and immune systems [2].

Undisturbed ovarian development during fetal/neonatal life is essential for reproductive success. Initially, primordial follicles consisting of the oocytes surrounded by pre-granulosa cells are formed from oocyte nests to establish the follicular pool – the only source of follicles throughout the entire female reproductive life-span [3]. In pigs, the oocyte nest breakdown and the formation of the reserve of primordial follicles are completed around post partum day 25 [4]. The transition of primordial follicles into primary follicles is mainly coordinated by oocyte-derived factors. The follicle development beyond the late primary stage requires additional bi-directional communication between the oocyte and granulosa cells as well as granulosa and theca cells [5]. The factors implicated in the regulation of early follicle development include the members of the transforming growth factor-β superfamily as well as steroid hormones, including androgens [6, 7].

Androgens act mainly via androgen receptors (ARs). The androgen-AR complex binds to androgen responsive elements of target genes recruiting co-regulators to affect the AR transactivation [8]. Androgens may also exert their action via non-genomic signalling which includes the activation of the PI3K/Akt pathway [9]. There is a growing evidence that androgens, in addition to their pivotal regulatory role in male reproduction, are also indispensable for proper development of ovaries [10]. The essential role of androgens in promoting early follicular growth in primates is well documented [11]. Previously, we have demonstrated the presence of androgen receptors in the porcine fetal and neonatal ovary [12] indicating the possible sites of androgen action. Our recent studies also demonstrated that antiandrogen flutamide induced changes in the ovarian gene expression, leading to delayed folliculogenesis in porcine fetuses [13, 14]. We have also found that the exposure to flutamide during the neonatal window affected early stages of folliculogenesis manifested by lower number of primordial follicles and higher number of early primary follicles [15]. Moreover, androgen deficiency induced by flutamide treatment during fetal and neonatal periods in pigs was found to affect their reproductive health in adulthood [16, 17]. These results emphasize the role of androgens in the regulation of porcine ovary function, indicating also the potential significance of environmental antiandrogens for the activation of ovarian AR.

To better understand molecular mechanisms underlying the flutamide action in the porcine ovary, RNA-seqencing (RNA-Seq) was employed to identify molecules important for early folliculogenesis. RNA-Seq is a sensitive and sophisticated technique allowing to identify not only the protein-coding transcripts, but also the non-coding transcripts including long non-coding RNAs (lncRNAs). LncRNAs are involved in the regulation of genes as well as in the control of mRNA processing and stability [18]. Moreover, heterogeneous lncRNAs may act as co-regulators for many transcription factors, including steroid hormone receptors [19]. To expand our knowledge on flutamide action in the ovary, we attempted to examine the role of lncRNAs in controlling gene expression. This approach should improve our understanding of mechanisms responsible for androgen-regulated gene expression in the neonatal porcine ovary. Therefore, in the current study, we analyzed the effects of flutamide on the expression of both, coding transcripts (genes) and lncRNAs.

## Methods

### Experimental design and sample collection

The experiment was conducted in accordance with the national guidelines and approved by the Local Ethics Committee at the Jagiellonian University in Krakow, Poland (approval number 150/2013, 187/2014 and 188/2014). Surgical procedures were performed by a veterinarian. In the present study, six pig neonates (Large White × Polish Landrace) from different litters were randomly allocated into two groups. Animals of the first group (*n* = 3) were injected s.c. daily with flutamide (FLU; Sigma-Aldrich, St. Louis, MO, USA) between postnatal days 1 and 10. Flutamide was suspended in corn oil and administered at a dose of 50 mg/kg body weight. This dose was chosen on the basis of our previous experiments [13, 14] as well as literature review [20, 21] to effectively antagonize androgen action without exerting any toxic effects in the pigs. Animals in the second group (*n* = 3) served as controls (CTR) and were given a vehicle only (corn oil). On the day after the last injection (postnatal day 11), both ovaries from the two groups were excised and one gonad was snap frozen in liquid nitrogen for RNA isolation, while the contralateral gonad was fixed in Bouin’s solution. All piglets were housed with theirs mothers and siblings during the entire experiment.

### Total RNA isolation and evaluation of RNA integrity

Total RNA was extracted from the collected ovaries using TRI Reagent solution (Ambion, Austin, TX, USA) according to the protocol of the manufacturer. RNA concentration and quality were determined spectrophotometrically (NanoVue Plus, GE Healthcare, Little Chalfont, UK) and RNA integrity was evaluated by microfluidic electrophoresis using a 2100 Bioanalyzer with RNA 6000 Nano LabChip kit (Agilent Technologies, Santa Clara, CA, USA). Only samples with RNA integrity number (RIN; 28 S/18 S ratio) above 7.5 were used for RNA-Seq (*n* = 3 for each group).

### Construction and sequencing of Illumina cDNA libraries

Depleted RNA obtained from 400 ng of total RNA was used to construct cDNA libraries (TruSeq Stranded mRNA Sample Prep Kit; Illumina, San Diego, CA, USA). Following RNA purification and fragmentation, first and second cDNA strands were synthesized. Next steps included 3′ ends adenylation, adapter ligation and library amplification (PCR). Quantification of the cDNA library templates was performed using KAPA Library Quantification Kit (KapaBiosystem, Wilmington, MA, USA). Library profiles were estimated using the DNA High Sensitivity LabChip kit on the 2100 Bioanalyzer (Agilent Technologies). Afterwards, libraries were sequenced on a NextSeq500 high throughput sequencing instrument (Illumina) with 150 paired-end sequencing (Sequencing No. 1 - Seq 1). Since the obtained data showed that the transcriptome of one control ovary (CTR3) differed clearly from other control ovaries, the RNA sequencing of all samples was repeated to eliminate errors of the sequencing procedure (Sequencing No. 2 - Seq 2). Consequently, we have analyzed transcripts from ovaries of three control (CTR1, CTR2, CTR3) and three flutamide-treated (FLU1, FLU2, FLU3) piglets, where each piglet represented a biological sample. Each biological sample, in turn, was represented by two technical samples originating from Seq 1 (CTR1a, CRT2a, CTR3a, FLU1a, FLU2a, FLU3a) and Seq 2 (CTR1b, CRT2b, CTR3b, FLU1b, FLU2b, FLU3b). Results of the Seq 2 confirmed that the CTR3 sample displayed a different transcriptome profile, and this sample was excluded from further analysis. Thus, the entire analysis of transcript expression level was performed on 4 control samples (2 biological samples) and 6 flutamide-treated samples (3 biological samples).

### Bioinformatic analysis of gene expression

The sequencing data from this study have been submitted (http://www.ncbi.nlm.nih.gov/sra) to the NCBI Sequence Read Archive (SRA) under accession No. BioProject ID: PRJNA413646. The identified lncRNA sequences have been deposited in the GenBank (MG014013 - MG014182). The quality of cDNA fragments obtained after each sequencing (raw reads) was first evaluated using FastQC v0.11.5 program (http://www.bioinformatics.babraham.ac.uk/projects/fastqc/). Next, the reads were trimmed using Trimmomatic v0.32tool [22] to remove from the dataset any remaining Illumina adapter sequences and to receive short reads (~ 90 nt). In addition, PHRED33 score was used to eliminate the sequences of average score lower than 20. Then, the trimmed fragments were mapped to the whole porcine genome (Sus_scrofa.Sscrofa10.2; Ensembl database with annotation version 10.2.85) using STAR v2.4.0.1 [23] and StringTiev1.0.4 [24, 25]. The latter tool together with Cufflinks v2.2.1 [26] allowed us to estimate the expression of both annotated and not annotated transcripts.

The RNA-Seq differential expression analysis of control (CTR1 and CTR2) and flutamide-treated (FLU1, FLU2 and FLU3) samples was done using Cuffdiff (10.1038/nbt.2450) and DESeq2 [27] combined with SVA [28] batch effect normalization using Bioconductor [29, 30] in R statistical software. By using such approach we attempted to eliminate the effect of sequencing. In the current study, the expression levels of both non-coding and coding transcripts (further referred to as transcripts) as well as the expression levels of coding transcripts only (further referred to as genes) were compared between flutamide treated and control samples. To identify differentially expressed transcripts (DETs) and differentially expressed genes (DEGs), the following criteria were applied (*P*-adjusted < 0.05 and absolute normalized (log_2_) fold change was higher or equal to 1) in both Cufflinks and DESeq2 plus SVA.

### Functional enrichment analysis (GO and KEGG pathway)

To analyze functions of differentially expressed genes (DEGs) and their involvement in biological processes, the DEGs were classified into categories of the Gene Ontology (GO) database. GO enrichment analysis was performed using g:Profiler tool [31] with *P*-value threshold 0.05. Moreover, to investigate the possible gene association networks between DEGs, the Bioinformatics Database STRING 10.5 (Search Tool for the Retrieval of Interacting Genes, http://string-db.org) was used [32]. The searching criteria were based on the co-occurrence of genes/proteins in scientific texts (text mining), co-expression and experimentally observed interactions. This analysis generated gene/protein interaction networks, where strength of the interaction score was set as 0.4.

### Identification, characterization and analysis of lncRNAs

**Fig. 1:**
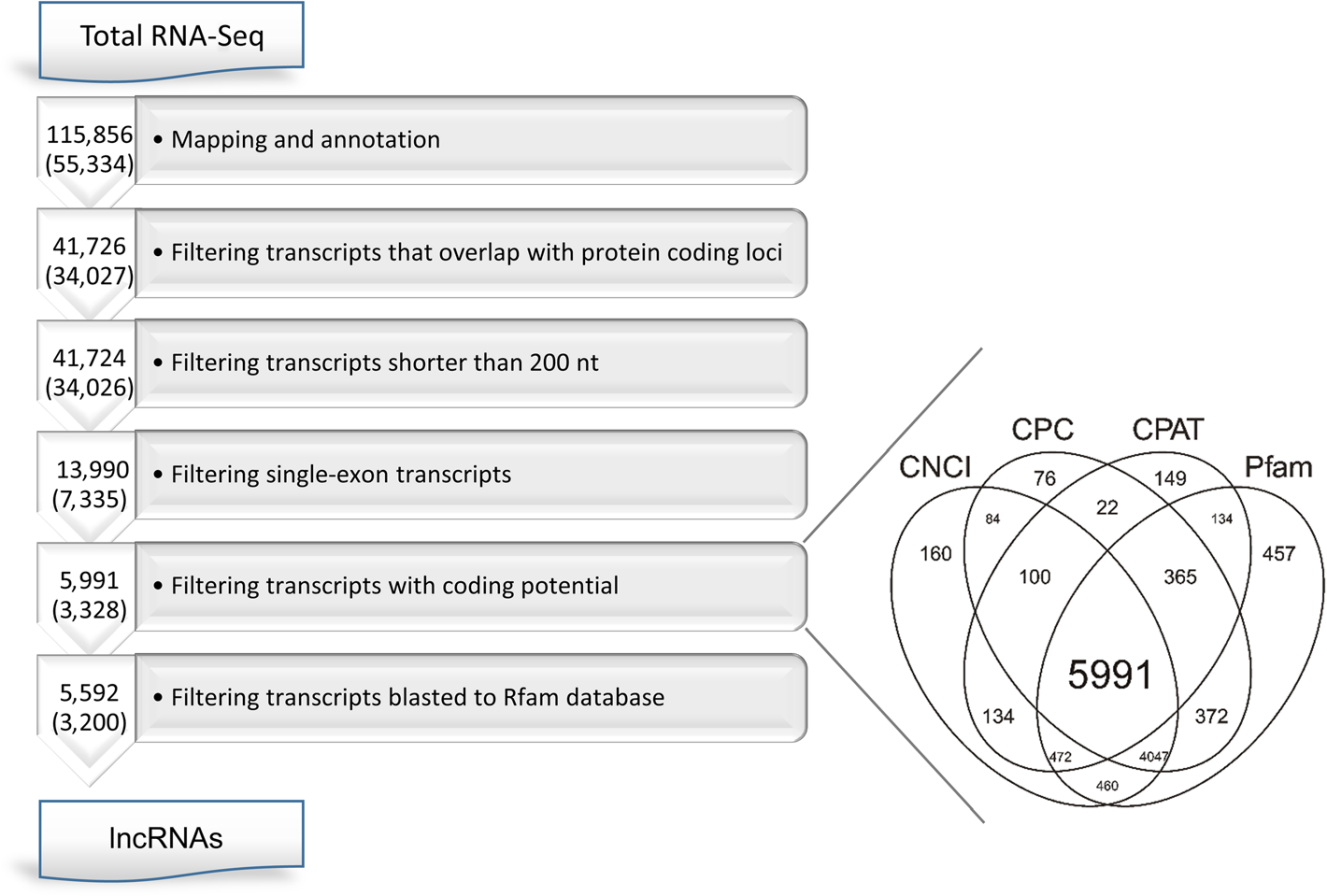
An overview of the stringent filtering pipeline used to identify 5,592 lncRNAs. Arrows contain the numbers of transcripts (TCONs) that passed a respective filter; the numbers of loci (XLOCs) are shown in brackets. Boxes describe actions that were employed at each step of the filtering procedure. Venn diagram shows the results obtained by using four different tools (CNCI, CPC, CPAT, Pfam) to filter out transcripts with coding potential

To identify known lncRNAs, the assembled transcripts were annotated with the use of GENCODE database. The customized multi-step pipeline was employed to identify putative novel lncRNAs in the ovaries of porcine neonates (Fig. 1). Briefly, protein-coding transcripts, transcripts with a single exon and those shorter than 200 nt as well as transcripts with coding potential were removed from the sequencing data, yielding novel lncRNAs. The total identified lncRNAs include both the novel transcripts and the previously annotated lncRNAs. The genomic features of the identified lncRNAs were characterized and compared with those of mRNAs (Welch’s *t*-test, *P* < 0.05). Next, the Cuffnorm (version 2.2.1) in the Cufflinks package was employed to normalize the identified lncRNA sequences to FPKM (fragments per kilobase of transcript per millions fragments sequenced) values. Next, the expression level of each lncRNA identified in the ovarian samples of flutamide-treated neonates (expressed in FPKMs) was compared to the expression level of the respective lncRNA identified in the control samples. Such approach allowed to identify differentially expressed lncRNAs (DELs; *P*-adjusted < 0.05 and log_2_ fold change (log_2_FC) ≥ 1.0; Cuffdiff software). In addition, similarity measure, which combines elements from Pearson correlation and Euclidean distance, was performed between the identified DELs and DEGs.

### Real-time PCR

To validate the RNA-Seq results, the expression level of several selected up- and down-regulated DEGs and DELs was confirmed by quantitative real-time PCR (qRT-PCR). The same templates were used in RNA-Seq and qRT-PCR. For cDNA synthesis, 1 μg of RNA was reverse transcribed using a High-Capacity cDNA Reverse Transcription Kit (Applied Biosystems, Foster City, CA, USA) according to the manufacturer’s protocol. The 20 μL total reaction volume contained random primers, dNTP mix, RNAse inhibitor and Multi Scribe Reverse Transcriptase. The reaction was performed in a Veriti Thermal Cycler (Applied Biosystems) according to the following thermal profile: (1) 25 °C for 10 min, (2) 37 °C for 120 min, and (3) 85 °C for 5 min. Real time PCR was performed using TaqMan Gene Expression Master Mix (Applied Biosystems) and porcine-specific TaqMan Gene Expression Assay (Applied Biosystems) for: zona pellucida glycoprotein 4 (*ZP4*; Ss03391382_m1), cytochrome P450 11A1 (*CYP11A1*; Ss03384849_u1), serpin A1 (*SERPINA1*; Ss03394873_m1), and lncRNA- TCONS_00107335 (Custom Assay AI39TQV; primer sequences: forward: CGGGAGAAACATTAATCCCTGTTTTAGA, reverse: TGATCTAGAGGTAACGAGAAACACTGT; probe sequence: TCATCTTGCTGTCAATAAA) with endogenous control for glyceraldehyde-3-phosphate dehydrogenase (*GAPDH*, Ss03373286_u1) following manufacturers’ instructions. All real-time PCR experiments were performed in triplicate, and non-template control was included in each run. The amplifications were performed with the StepOneTM Real-Time PCR System (Applied Biosystems) according to the recommended cycling program (2 min at 50 °C, 10 min at 95 °C, 40 cycles of 15 s at 95 °C, and 1 min at 60 °C). Threshold cycles (Ct values) for the expression of each gene were calculated using StepOne software. The relative mRNA expression level was calculated using the 2^-ΔΔCt^ method [33], adjusting the ZP4, CYP11A1, SERPINA1 and TCONS_00107335 expression to the expression of GAPDH. Data were expressed as the overall mean ± SEM. Statistical analysis was performed using Statistica v.13.1 program (StatSoft, Inc., Tulsa, OK, USA). The nonparametric Mann-Whitney U-test was used to determine significant differences between the control and flutamide-treated groups. The differences were considered statistically significant at the 95% confidence level (*P* < 0.05).

## Results

### The effects of flutamide on the transcriptome of the porcine neonate ovaries

Sequencing of mRNA isolated from porcine neonatal ovaries produced 24,265,964 –33,066,267 raw reads per sample. After rejecting low quality reads (PHRED< 20), the remaining reads (22,025,084 – 29,051,215 per sample) were mapped to the annotated whole porcine genome (Sus_scrofa.Sscrofa10.2). The percentage of the reads aligned to the genome ranged from 71.45% to 78.81%, and an average of 75.47% of these reads were mapped to a unique location. The total number of the transcripts identified in the piglet ovaries ranged from 40,724 to 44,969 per ovary. The summary of data obtained from RNA-Seq of porcine neonatal ovaries is presented in Table 1.

**Table 1:** Summary of data obtained from RNA-Seq of porcine neonatal ovaries

Sample	RNA Sequencing No.1						RNA Sequencing No.2					
	CTR1a	CTR2a	CTR3a	FLU1a	FLU2a	FLU3a	CTR1b	CTR2b	CTR3b	FLU1b	FLU2b	FLU3b
Number of row reads	25,218,729	24,265,954	26,786,205	29,657,600	31,499,003	33,066,267	25,684,749	29,793,840	24,982,931	26,909,112	26,958,364	29,883,752
Number of processed reads	22,946,350	22,025,084	23,919,783	26,265,118	28,431,555	29,051,215	24,815,278	28,489,892	23,409,293	25,573,491	25,420,566	28,108,743
% of processed reads	90.99	90.77	89.30	88.56	90.26	87.86	96.61	95.62	93.70	95.04	94.30	94.06
Unique reads												
Number of uniquely mapped reads	16,920,873	15,737,185	17,549,085	19,080,540	20,774,178	20,886,099	19,415,040	22,389,585	18,221,092	20,157,124	19,841,031	21,895,703
% of uniquely mapped reads	73.74	71.45	73.37	72.65	73.07	71.89	78.24	78.59	77.84	78.82	78.05	77.90
Average mapped length, nt	176.97	176.39	177.02	176.83	176.94	176.78	177.53	177.65	177.56	177.60	177.68	177.56
Multi-mapping reads												
Number of reads mapped to multiple loci	1,728,713	1,588,546	1,774,327	1,896,456	2,078,804	2,120,266	1,905,286	2,239,355	1,818,916	1,987,466	1,973,831	2,205,331
% of reads mapped to multiple loci	7.53	7.21	7.42	7.22	7.31	7.30	7.68	7.86	7.77	7.77	7.76	7.85
Identified transcripts												
Number of identified transcripts	43,478	43,702	41,124	42,171	43,952	43,531	42,672	44,969	40,724	41,812	43,259	43,307
% of identified transcripts	71.85	72.22	67.96	69.69	72.63	71.93	70.51	74.31	67.29	69.09	71.48	71.56

*FLU* ovaries from flutamide-treated piglet, *CTR* ovaries from NaCl-treated piglet

Each biological sample was sequenced two times (for details please see the M & M section)

Sequencing No. 1 (CTR1a, CTR2a, CTR3a, FLU1a, FLU2a and FLU3a) and Sequencing No. 2 (CTR1b, CTR2b, CTR3b, FLU1b, FLU2b and FLU3b) produced two

separate sets of data. The statistics are presented for each sample separately

Preprocessing of data included clipping of adapters, trimming of read ends and removing low quality reads

“Unique reads” refer to reads that were mapped to a unique (only one) location of the reference genome

“Multi-mapping reads” refer to reads aligned to more than one locus on the reference genome

Technical samples: CTR1a and CTRb1, FLU1a and FLU1b etc

**Fig. 2:**
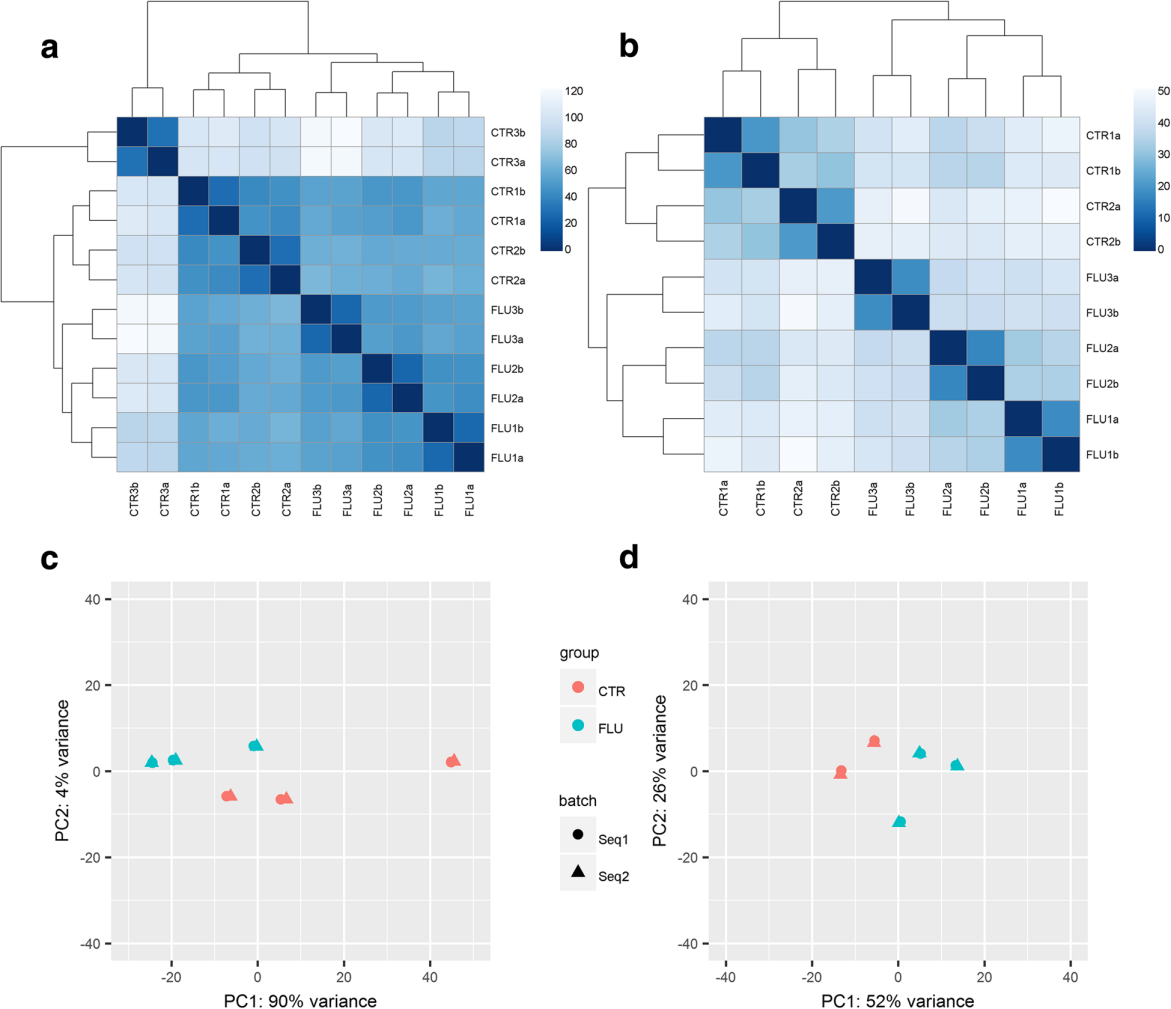
Distance matrices (**a**, **b**) and PCA analysis (**c**, **d**) illustrating the transcriptomic profile of the ovaries of pig neonates treated with flutamide. The color scale represents the distances between biological replicates, where the most dark blue stands for the smallest distance. **a**, **c**/ These plots show results obtained from the analysis of three control (CTR1, CTR2, CTR3) and three flutamide-treated (FLU1, FLU2, FLU3) piglets, where each piglet represents a biological sample. Each biological sample is represented by two technical samples originating from Seq1 (**a**) and Seq2 (**b**) which produces 12 samples; − CTR1a and CTR1b, CTR2a and CTR2b, CTR3a and CTR3b, FLU1A and FLU1b, FLU2A and FLU2b, FLU3A and FLU3b (6 CTR vs. 6 FLU); **b**, **d**/ These plots show results obtained from the analysis of two control (CTR1, CTR2) and three flutamide-treated (FLU1, FLU2, FLU3) piglets – one control piglet was removed from the analysis due to the clearly distinct transcriptomic profile (4 CTR vs. 6 FLU, for details please see the M&M and Results sections and Fig. 2)

**Fig. 3:**
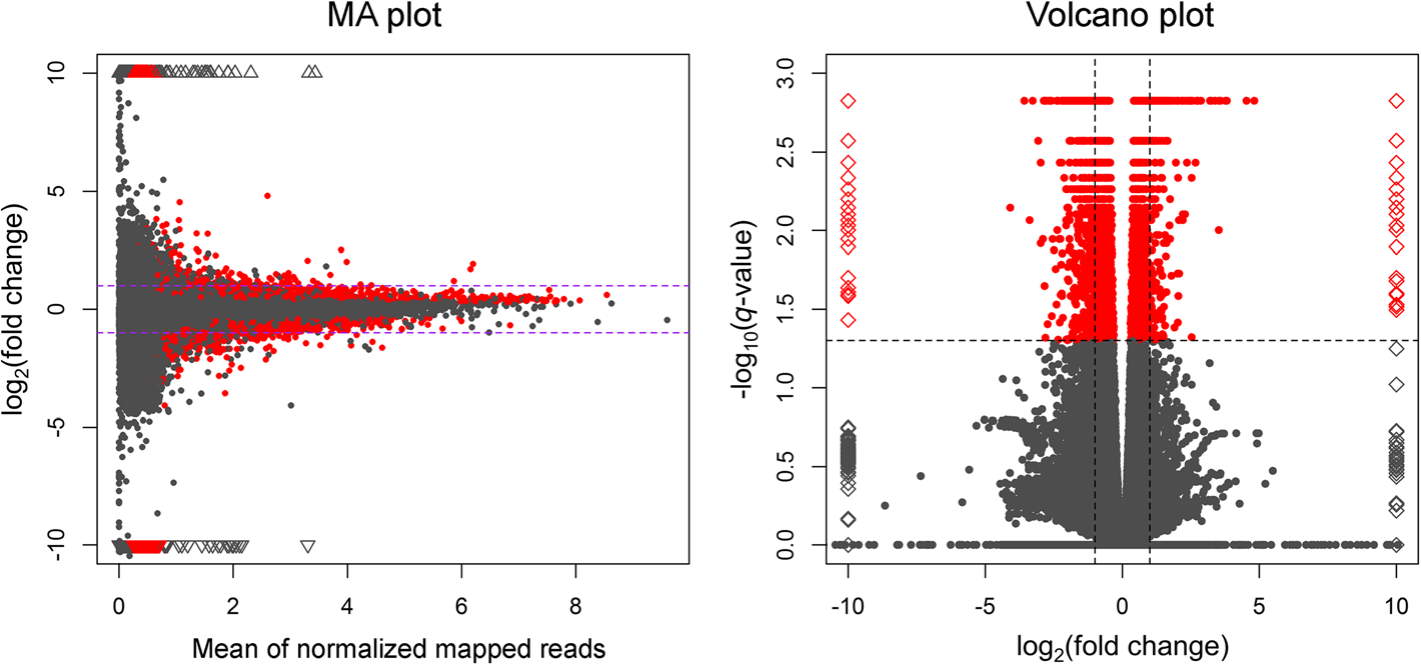
Distribution of transcripts presented as MA plot and Volcano plot determined by Cufflinks. Red points represent differentially expressed transcripts (DETs; *P*-adjusted < 0.05). Triangles and diamonds represent transcripts with the expression level out of the plot scale. Horizontal lines in the MA plot and vertical lines in the Volcano plot indicate the thresholds of log_2_FC = 1 and log_2_FC = − 1

As shown in Fig. 2a and c, genes of one of control ovaries (CTR3) clustered separately from those of other control ovaries, which disposed us to remove this sample from further analysis. The analysis of distance matrices revealed a high level of similarity between biological replicates of the remaining control samples as well as between replicates of the flutamide-treated samples (Fig. 2b and d). Distribution of transcripts, including DETs, (*P*-adjusted < 0.05, log_2_FC ≥ 1.0) in the ovaries of porcine neonates treated with flutamide are visualized in Fig. 3.

**Fig. 4:**
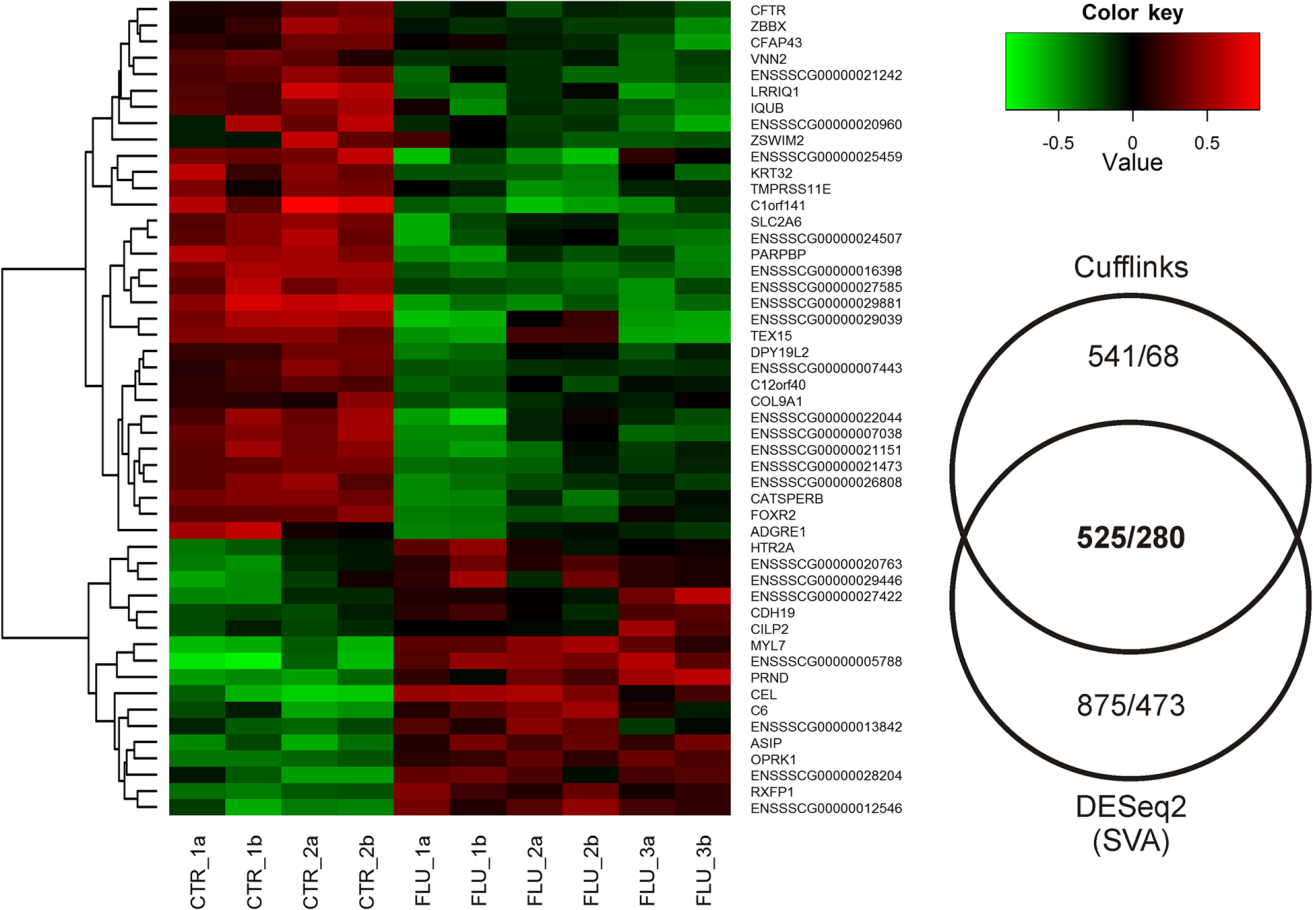
Differentially expressed genes (DEGs; *P*-adjusted < 0.05 and log_2_ fold change ≥1.0) in the ovaries of porcine neonates treated with flutamide. The left panel shows a heatmap illustrating the expression profile of top 50 DEGs: the red blocks represent up-regulated genes, and the green blocks represent down-regulated genes; the color scale of the heatmap represents the expression level, where the most bright green stands for − 1.0 log_2_ fold change and the most bright red stands for 1.0 log_2_ fold change. The selection of the top 50 DEGs was based on the least standard deviations within a group (CTR and FLU). The right panel presents the number of differentially expressed transcripts (DETs) and DEGs (DETs/DEGs) obtained by employing two statistical tools, i.e., Cufflinks and DESeq combined with the SVA batch normalization effect

A total of 525 DETs and 280 DEGs were determined in the current study. We identified 176 up- and 104 down-regulated DEGs in neonate ovaries after the flutamide treatment. The expression profile of the top 50 DEGs (i.e., DEGs with the highest absolute log_2_FC values) is presented in Fig. 4. The log_2_FC value for DEGs ranged from − 4.09 (ENSSSCG00000001212) to 3.77 (ENSSSCG00000016799) (see Additional file 1). All up- and down-regulated genes identified in the ovaries of porcine piglets treated with flutamide are presented in Additional file 2.

### Functional classification of flutamide-affected genes

**Fig. 5:**
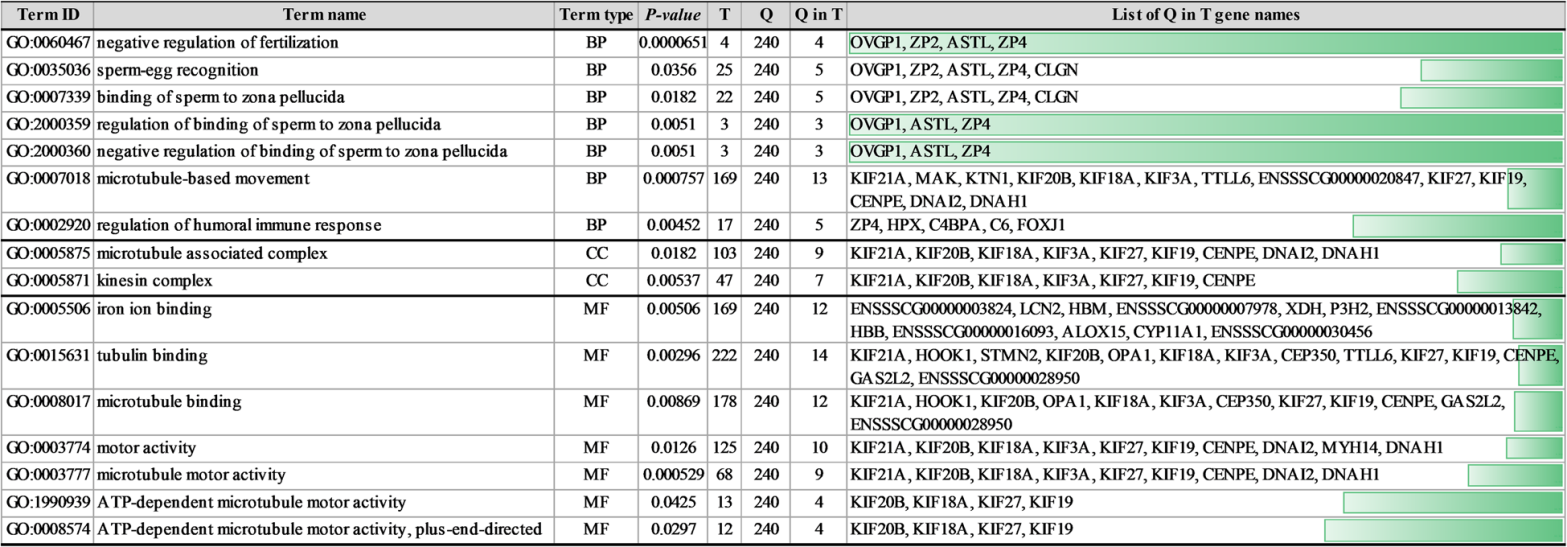
Results of Gene Ontology enrichment analysis of differentially expressed genes identified in the ovaries of porcine neonates exposed to flutamide. BP: Biological process, CC: Cellular component, MF: molecular function, T: number of genes ascribe to a particular term in GO database, Q: number of DEGs that are annotated in GO database, Q in T: Number of annotated DEGs that are ascribed to a particular term

**Fig. 6:**
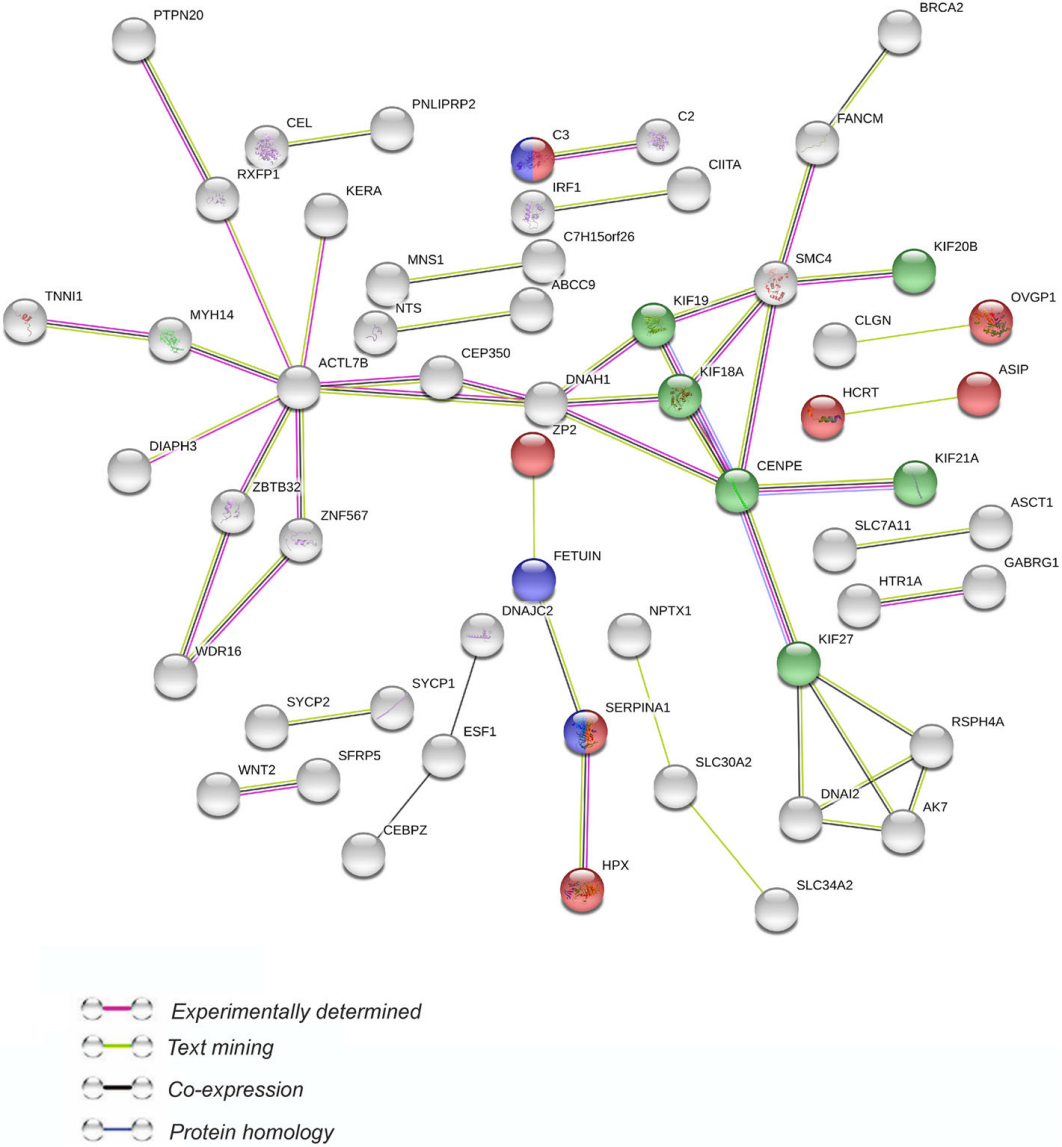
STRING-generated interaction network of the differentially expressed genes (DEGs) in the porcine ovaries after flutamide administration during the neonatal period. STRING v10.5 was used to derive the network of 280 DEGs applying following prediction methods: text mining (connecting green links), co-expression (connecting black links), experimentally observed interactions (connecting red links), protein homology (connecting blue links). The nodes that did not interact with other nodes were deleted. The color of the nodes illustrate the assignment to the appropriate GO categories: red – biological processes, blue – molecular function and green – cellular component. Full names of the presented DEGs are listed in Additional file 1. Interaction score > 0.4; protein-protein interaction enrichment *P*-value = 0.000237

To indicate possible functions of DEGs identified in the ovaries of flutamide-treated porcine neonates, the genes were classified into three main categories (“biological process”, “molecular function” and “cellular component”) according to GO database. Forty one genes out of 280 DEGs were assigned to GO terms (Fig. 5). The DEGs classified into “biological process” were mainly annotated to processes included “microtubule-based movement” (13 genes), “sperm-egg recognition” (5 genes) and “binding of sperm to zona pellucida” (5 genes). The “molecular function” GO category encompassed, among others, DEGs annotated to: “tubulin binding” (14 genes), “iron ion binding” (12 genes) and “microtubule binding” (12 genes; Fig. 5). The “cellular component” category linked the DEGs to: “microtubule associated complex” (9 genes) and “kinesin complex” (7 genes; Fig. 5). To inquire into the role of DEGs in the ovarian response to flutamide, we analyzed these genes by means of STRING v10.5 tool. The analysis generated a gene/protein interaction network (Fig. 6; the strength of interaction score > 0.4) consisting of 56 DEGs (nodes) and 50 edges. Nodes devoid of any interactions were deleted from the network. Seven DEGs were classified into “biological processes”, three genes were annotated to “endopeptidase inhibitor activity”, and six genes were linked to the “PFAM kinesin motor domain family”. *ACTL7B* (actin like 7B, 8 edges), and *CENPE* (centromere-associated protein E, 6 edges) were the most interacting nodes. The strongest interactions (expressed as the number of links found between any two genes) were identified between *CENPE, KIF18A* (kinesin family member 18A)*, KIF19* (kinesin family member 19)*, SMC4* (structural maintenance of chromosome 4) and *DNAH1* (dynein axonemal heavy chain 1) (Fig. 6).

### Identification and characterization of lncRNAs in the ovarian transcriptome of porcine neonates

**Fig. 7:**
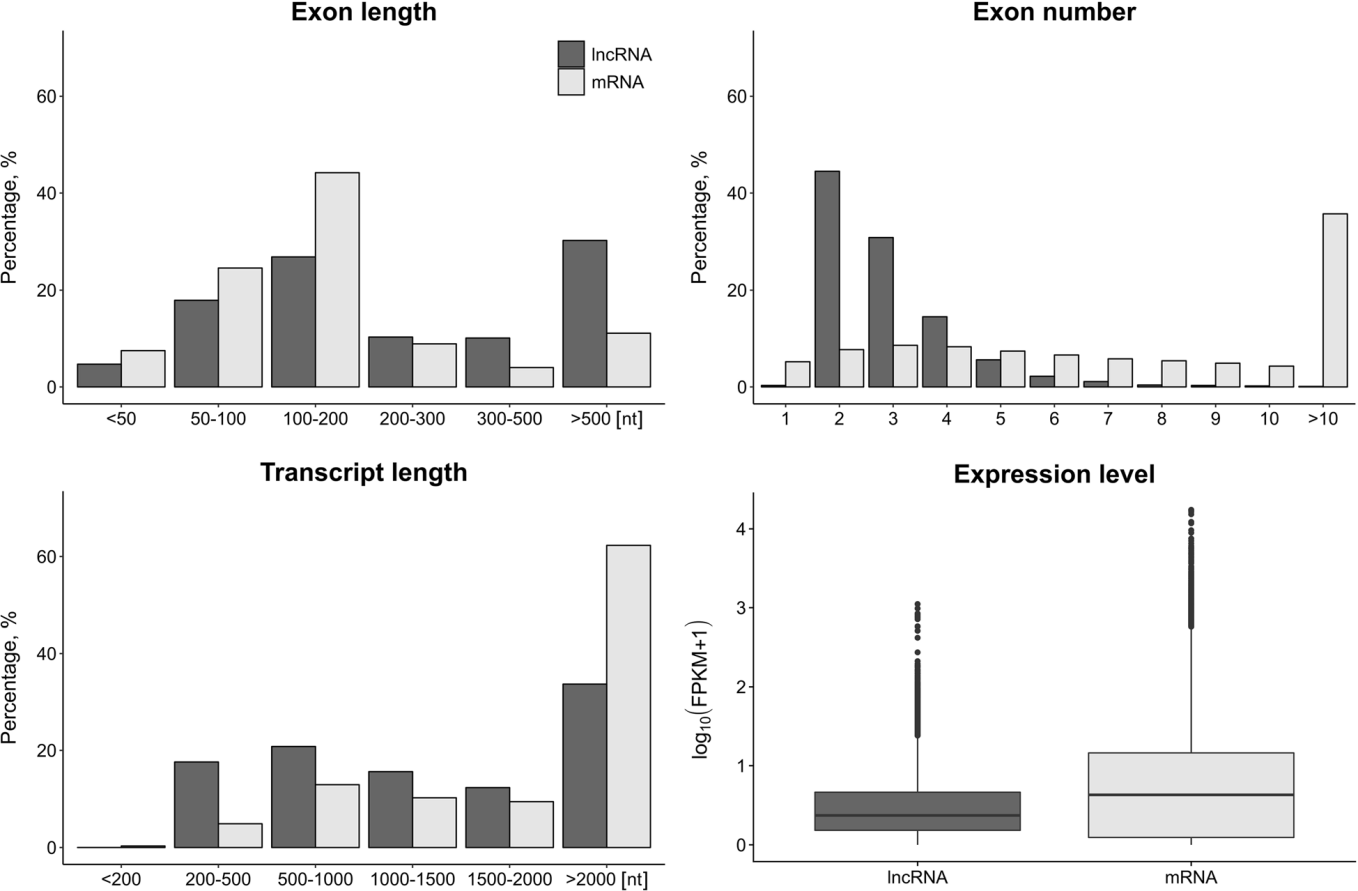
The comparison of genomic features and the expression level of lncRNAs and mRNAs. The lncRNA and mRNA transcripts were compared in respect to transcript and exon length as well as exon number. The numbers over bars depict the exact values of exon length, exon number and transcript length. The expression level of lncRNAs and mRNAs is presented as boxplot (median and quartiles)

The customized multi-step identification pipeline was applied to distinguish lncRNAs from all other assembled transcripts (Fig. 1). A total of 5,592 RNA sequences were identified as lncRNAs, including 136 lncRNAs (located in 53 long non-coding loci) already annotated in databases. Transcript length, exon length, exon number and expression level were compared between the identified lncRNAs and mRNAs (Fig. 7). The average transcript and exon length of lncRNAs were 1992 nt (173–25,353 nt) and 669 nt (11–25,163 nt), respectively. The average exon number of lncRNAs and mRNAs was 2.97 and 10.38, respectively (Fig. 7).

The average length of lncRNA transcripts was shorter and lncRNA exon length was longer (*P* < 0.001) than those of mRNAs (1992 vs. 3651 nt and 669 vs. 351 nt, respectively). 30% of lncRNAs and 60% of mRNAs were longer than 2000 nt. The exon length of more than 40% of mRNA transcripts ranged from 100 to 200 nt and 25% of lncRNAs ranged from 100 to 200 or > 500. The mean exon number of lncRNAs was lower (*P* < 0.001) than that of mRNAs. Most lncRNAs contained two exons, while most mRNAs had more than ten exons. The average lncRNAs expression level (log_10_FPKM ~ = 0.5) was lower than that of mRNA (log_10_FPKM ~ = 0.84) (*P* < 0.001; Fig. 7).

The effects of flutamide on the lncRNA expression profile in the ovaries of porcine neonates.

**Fig. 8:**
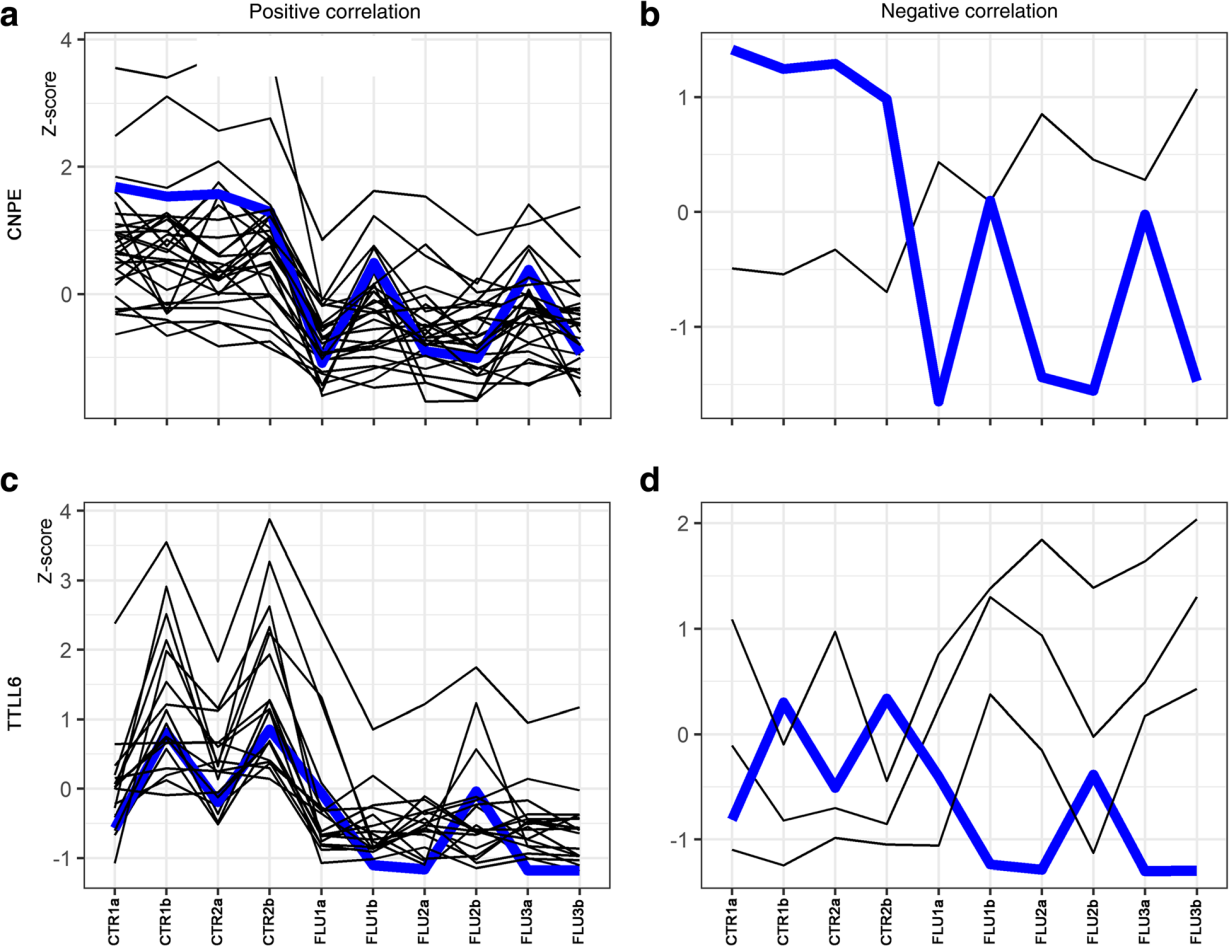
The effects of correlation analysis performed between two exemplary DEGs (**a**, **b**/ *CENPE* – centromere-associated protein E, and **c**, **d**/ *TTLL6* - tubulin tyrosine ligase like 6) and DELs. The expression of DEGs was analyzed in the ovaries of porcine neonates treated with flutamide by Bioconductor in R package. Expression data are presented as normalized values (Z-scores). Plots for analyzed DEGs are depicted as blue lines and for correlated DELs as black lines

A total of 98 differentially expressed lncRNAs (DELs; *P-*adjusted < 0.05 and log_2_FC ≥ 1.0) were identified in the current study (see Additional file 3). The correlations found between 280 DEGs and 98 DELs identified in the ovaries of porcine neonates treated with flutamide are depicted in Additional file 1 and visualized in a form of heatmap in Additional file 4. The graphical illustration of positive and negative correlations between two exemplary DEGs (*CENPE* and *TTLL6* – tubulin tyrosine ligase like 6) and DELs is shown in Fig. 8. The log_2_FC values for DELs ranged from − 3.38 (XLOC_052195) to 2.79 (XLOC_033570). Twenty nine out of 98 DELs identified in the ovaries were up- and 69 were down-regulated. The expression profile of the identified up- and down-regulated lncRNAs is presented in Additional file 5.

To learn how the lncRNAs may affect the ovarian response to flutamide, their potential target genes were identified. Functional analysis showed that the identified target genes were enriched in 16 GO terms (seven in “biological process”, seven in “molecular function” and two in “cellular component”). Majority of the genes were related with “tubulin binding” (GO:0015631), “microtubule-based movement” (GO:0007018), “microtubule binding” (GO:0008017) and “iron ion binding” (GO:0005506). Kinesin family genes (*KIF3A, KIF18A*, *KIF21A*, *KIF20B*) and genes associated with microtubule dynamics (*HOOK1*, *CENPE*) were found among these genes. The expression of target genes listed in the previous sentence was down-regulated by flutamide similar to the expression of the lncRNAs potentially involved in the regulation of these genes (i.e., the expression of lncRNAs and the expression of their target genes were positively correlated).

### Validation of selected DEGs and DELs by qRT-PCR

**Fig. 9:**
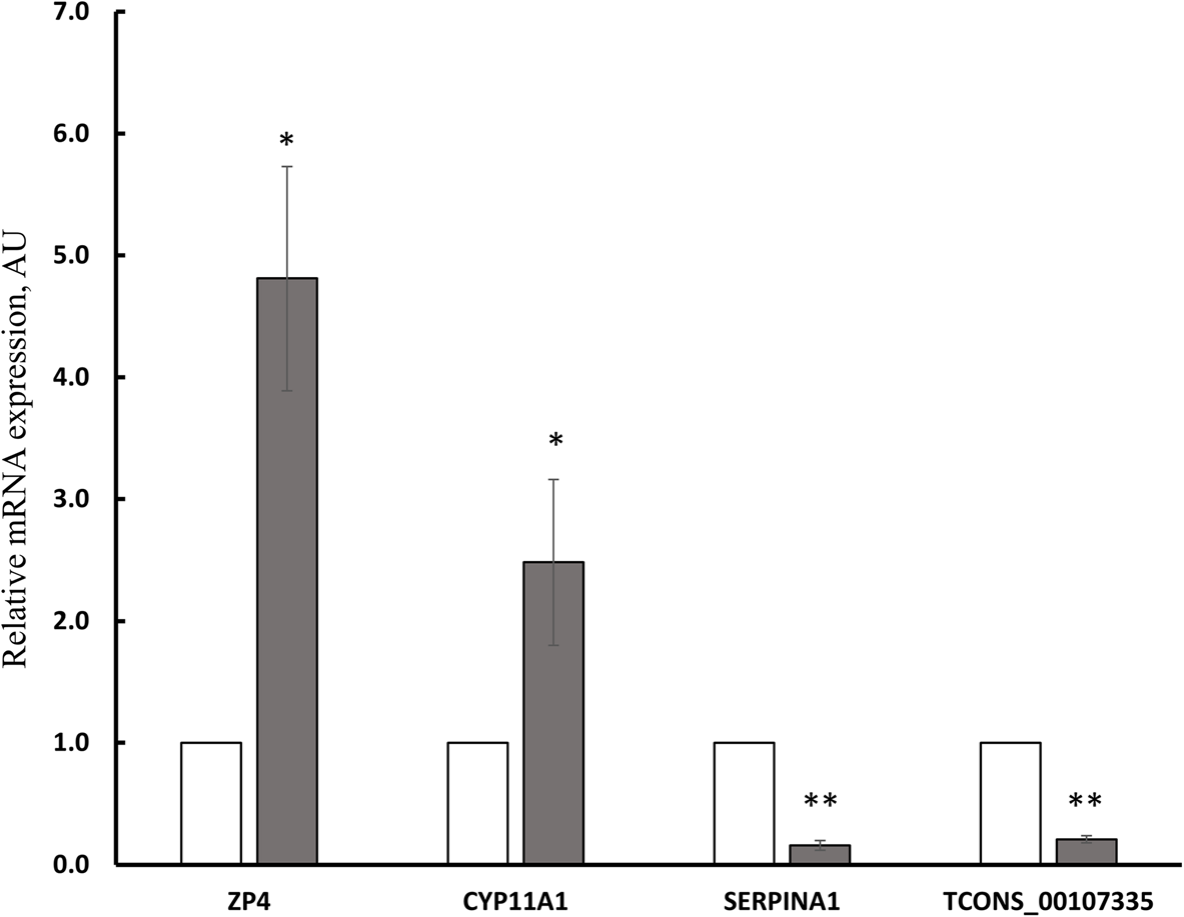
Quantitative real-time PCR of the selected differentially expressed genes (DEGs) and lncRNAs (DELs) identified by RNA-Seq in the ovaries from control (open bars) and flutamide-treated (shaded bars) neonatal pigs. The validation was performed using the same RNA samples as were used in RNA-Seq. The expression of the selected up- and down-regulated genes was presented as 2^-ΔΔCt^ relative to *GAPDH*. Data were expressed as mean ± SEM. Asterisks designate significant differences between control and flutamide-treated groups (Mann-Whitney U test; *P < 0.05, **P < 0.01). *ZP4*: zona pellucida glycoprotein 4, *CYP11A1*: cytochrome P450 11A1, *SERPINA1*: serpin A1, TCONS_00107335; AU: arbitrary units

To validate RNA-Seq data, three DEGs, i.e., zona pellucida glycoprotein 4 (*ZP4*)*,* cytochrome P450 11A1 (*CYP11A1*), and serpin A1 (*SERPINA1*) as well as one DEL (TCONS_00107335), were submitted to quantitative RT-PCR. These transcripts were selected based on their potential importance for the ovary functions as well as the high log_2_FC value. The expression of the selected DEGs and DEL confirmed the results obtained by RNA-Seq (Fig. 9).

## Discussion

Recently, we have demonstrated that androgen deficiency induced by flutamide treatment during fetal life led to delayed formation of primordial follicles as well as their delayed transition into primary stage within the fetal porcine ovary [13, 14]. We have also found that the exposure to flutamide during the neonatal window decreased ovarian cell proliferation, which probably was partially responsible for the restricted development of early primary follicles in the neonate ovaries [15]. These findings contributed to better understanding of androgen involvement in the promotion of follicle formation and development. In the current study, RNA-Seq has been employed to extend our knowledge on the role of androgens in neonatal folliculogenesis as well as to examine the impact of the chemical compound displaying anti-androgenic activity on ovarian function.

We were able to identify a number of differentially expressed genes (DEGs) and lnRNAs (DELs) in the ovarian transcriptome of the 11-day-old piglets treated neonatally with flutamide. LncRNAs have recently become recognized as molecules involved in the regulation of a wide range of biological processes including epigenetic regulation, chromatin modification, genomic imprinting, transcriptional control and pre−/post-translational mRNA processing [34, 35]. An increasing evidence links the dysregulation of lncRNA expression with the promotion of tumors including epithelial ovarian cancer [36, 37]. Little is known, however, about the significance of lncRNAs in the regulation of early follicle development.

Gene Ontology database analysis of the 280 identified DEGs revealed that some DEGs were classified into “biological processes” categories related to mechanisms regulating sperm-zona pellucida interaction. Moreover, the “molecular function” and “cellular components” categories linked DEGs to functions associated with cellular transport, cell divisions and cytoskeleton. In addition, STRING software demonstrated the strongest interactions between *CENPE*, *KIF18A*, *KIF19*, *SMC4* and *DNAH1*, i.e., genes related to cell proliferation.

Some of the down-regulated DEGs may be involved in the regulation of ovarian cell proliferation in the neonatal pig. Proliferation of granulosa cells is prerequisite for the progression of follicular growth. Members of kinesin family are required for intracellular transport of molecules or organelles as well as for mitosis, including such processes as bipolar spindle assembly, chromosome alignment, chromosome segregation and cytokinesis [38, 39]. The essential role of KIF18A in the regulation of chromosome congression during pro-metaphase and chromosome alignment during metaphase was previously reported [40, 41]. Moreover, RNAi-mediated KIF18A deficiency resulted in mitotic arrest in HeLa cells [40]. It was also demonstrated that KIF18A physically interacted with CENPE [42]. Interestingly, CENPE, which is required for efficient and stable microtubule capture at the kinetochore [43], was recognized in the present study (STRING software) as the most interacting node. Moreover, kinesin-2 complex (KIF3A/3B) was found to be localized in spindle microtubules and centrosomes of HeLa cells [44], and aberrant mitosis was demonstrated in the NIH3T3 cells transfected with a dominant-negative mutant of KIF3A/3B [44]. In the current study, *KIF3A*, *KIF18A* and *CENPE* were found to be down-regulated in the ovaries of 11-day-old piglets exposed to flutamide during neonatal period. *SMC4*, important for chromosome condensation and segregation, was downregulated in these ovaries, too. Zhang et al. [45] demonstrated that *SMC4* knockdown significantly inhibited the proliferation and invasion of A549 cells. Since flutamide reduced the number of proliferating granulosa cells in neonate piglets [15], we may conclude that this reduction may, at least partially, originate from the flutamide-induced down-regulation of *KIF3A*, *KIF18A*, *CENPE* and *SMC4*.

The follicular growth is a complex phenomenon and involves various metabolic and proteolytic events mediated by a number of enzymes [46]. In the current study flutamide affected the expression of serine protease inhibitors i.e., *SERPINA1*, *SERPINB5* and *SERPINA6*. These inhibitors are important in balancing the activity of proteases, which influence diverse biological processes, including cellular differentiation, apoptosis, extracellular matrix remodelling, fibrinolysis, coagulation, inflammation and cell mobility [47]. It has been reported that serpins are involved in follicular development of different species [48, 49]. In addition to its role in inflammation, SERPINA1 has also been shown to increase low density lipoprotein (LDL) binding/uptake and to upregulate LDL receptor level [50]. Since LDLs provide substrate for steroid hormone production, the flutamide-induced down-regulation of *SERPINA1* expression suggests that this antiandrogen may affect ovarian steroidogenesis in piglets. On the other hand, flutamide increased the expression of *CYP11A1*, an enzyme responsible for converting cholesterol into pregnenolone. We demonstrated previously the stage-dependent changes in the luteal CYP11A1 expression of pregnant pigs exposed to flutamide [51]. Regardless of the fact that currently it is not possible to clarify the relationships between flutamide, SERPINA1 and CYP11A1 with regards to steroidogenesis, the presented results support the notion that anti-androgens affect steroid production in the ovaries of neonatal piglets.

Mammalian oocyte is surrounded by zona pellucida (ZP), a transparent extracellular matrix playing an important role during the period between species-selective sperm-oocyte recognition and implantation [52]. Porcine ZP consists of glycoproteins ZP2, ZP3 and ZP4, but only the ZP3/ZP4 heterocomplex has sperm binding activity [53]. It was demonstrated that in mice ZP2 is the substrate for ovastacin, an oocyte-specific metalloendoprotease (encoded by *ASTL)* and component of cortical granules. Cleavage of ZP2 by ovastacin provides a definitive block to polyspermy [54]. Moreover, Sachdev et al. [55] reported a role of ovastacin in sperm-egg interaction prior to fusion and sperm internalization in the mouse. Ovarian expression of ovastacin was not only demonstrated to be conserved in mammals, but also limited to the oocytes of follicles at the primary-secondary transition [56]. In the current study, *ZP2*, *ZP4* and *ASTL* were up-regulated in the ovaries of 11-day-old piglets exposed to flutamide during the neonatal period. This may indicate that flutamide affects expression of genes involved in oocyte fertilization in the pig. Such notion is supported by meiotic abnormalities and fertilization failure observed in rat oocytes *in vitro* exposed to 2-hydroxyflutamide [57]. However, further research is necessary to uncover the nature of molecular processes underlying the association between neonatal anti-androgen exposure and oocyte fertilizing ability in pigs.

In the present study, along with coding transcripts, lncRNAs were also identified in ovarian tissue of porcine neonates. Ninety eight of the identified lncRNAs were differentially expressed in the ovaries of control and flutamide treated piglets. GO enrichment analysis of the genes targeted by the flutamide-affected lncRNAs revealed that a majority of the genes were associated with tubulin binding, microtubule-based movement and microtubule binding terms. This group of target genes includes those encoding “kinesin family members of motor proteins” important for intracellular transport and cell division [38, 39]. These findings clearly support the hypothesis concerning the link between the flutamide-induced inhibition of granulosa cell proliferation [15] and the changes in the expression of kinesins.

## Conclusions

In the current study, 280 differentially expressed genes were identified in the porcine ovaries after flutamide administration during the neonatal period. Flutamide was found to affect expression of *KIF3A*, *KIF18A*, *CENPE* and *SMC4* which may be responsible for the decrease in proliferation of ovarian cells and, in consequence, for changes in folliculogenesis. Moreover, the expression of genes involved in ovarian steroidogenesis and oocyte fertilization was also disturbed by flutamide, displaying the potential to affect female reproductivity in adulthood. In conclusion, the results obtained in the current study, by employing an antiandrogen flutamide as a research tool and with the assistance of RNA-Seq, confirmed the indispensable role of androgens during the early stages of folliculogenesis in the pig. In addition, they emphasized the importance of monitoring environmental pollutants with antiandrogen activity that may influence androgen-mediated processes within neonatal ovaries.

## Additional files

Additional file 1: An overview of up and down regulated genes [*P* < 0.05; (log_2_) fold change] in the ovaries of flutamide-treated piglets (XLS 95 kb)

Additional file 2: Heatmap illustrating the expression profile of all 280 differentially expressed genes (DEGs; *P*-adjusted < 0.05 and log_2_ fold change ≥1.0) in the ovaries of porcine piglets treated with flutamide. The red blocks represent up-regulated genes, and the green blocks represent down-regulated genes. The color scale represents the expression level, where the most bright green stands for − 2.0 log_2_ fold change and the most bright red stands for 2.0 log_2_ fold change. (PNG 68 kb)

Additional file 3: An overview of all differentially expressed lncRNAs [*P* < 0.05; (log_2_) fold change] in the ovaries of flutamide-treated piglets. (XLS 61 kb)

Additional file 4: Heatmap illustrating correlations found between 280 differentially expressed genes (DEGs; *P*-adjusted < 0.05 and log_2_ fold change ≥1.0) and 98 differentially expressed long non-coding RNAs (DELs; *P*-adjusted < 0.05 and log_2_ fold change ≥1.0) in the ovaries of porcine piglets treated with flutamide. The red color represents a positive correlation and the blue color represents a negative correlation. (TIF 1011 kb)

Additional file 5: Differentially expressed lncRNAs (DELs; *P*-adjusted < 0.05 and log_2_ fold change ≥1.0) in the ovaries of porcine neonates treated with flutamide. The left panel shows a heatmap illustrating the expression profile of all DELs: the red blocks represent up-regulated DELs, and the green blocks represent down-regulated DELs; the color scale of the heatmap represents the expression level, where the most bright green stands for − 2.0 log_2_ fold change and the most bright red stands for 2.0 log_2_ fold change. The right panel presents the number of DETs/DELs obtained by employing two statistical methods, i.e., Cufflinks and DESeq combined with SVA batch normalization effect. (TIF 461 kb)

## Abbreviations

ACTL7B: Actin like 7B
AR: Androgen receptor
ASTL: Ovastacin
CENPE: Centromere-associated protein E
CTR: Control
CYP11A1: Cytochrome P450 11A1
DEGs: Differentially expressed genes
DELs: Differentially expressed lncRNAs
DETs: Differentially expressed transcripts
DNAH1: Dynein axonemal heavy chain 1
EACs: Endocrine-active chemicals
FLU: Flutamide
GAPDH: Glyceraldehyde-3-phosphate dehydrogenase
GO: Gene Ontology database
KIF: Kinesin family member
KIF3A/3B: Kinesin-2 complex
LDL: low density lipoprotein
lncRNA: Long non-coding RNA
nt: Nucleotides
qRT-PCR: Real-time PCR
RIN: RNA integrity number
RNA-Seq: RNA sequencing
SEM: Standard error of mean
Seq: Sequencing
SERPIN: Serine protease inhibitor
SERPINA1: Serpin A1
SMC4: Structural maintenance of chromosome 4
SRA: Sequence Read Archive
TTLL6: Tubulin tyrosine ligase like 6
ZP: Zona pellucida glycoprotein
