# Supplementary figures and images for: Flutamide-induced alterations in transcriptional profiling of neonatal porcine ovaries
Source: J Anim Sci Biotechnol. 2019 Apr 3;10:35. doi: 10.1186/s40104-019-0340-y (PMC6446412; doi:10.1186/s40104-019-0340-y)

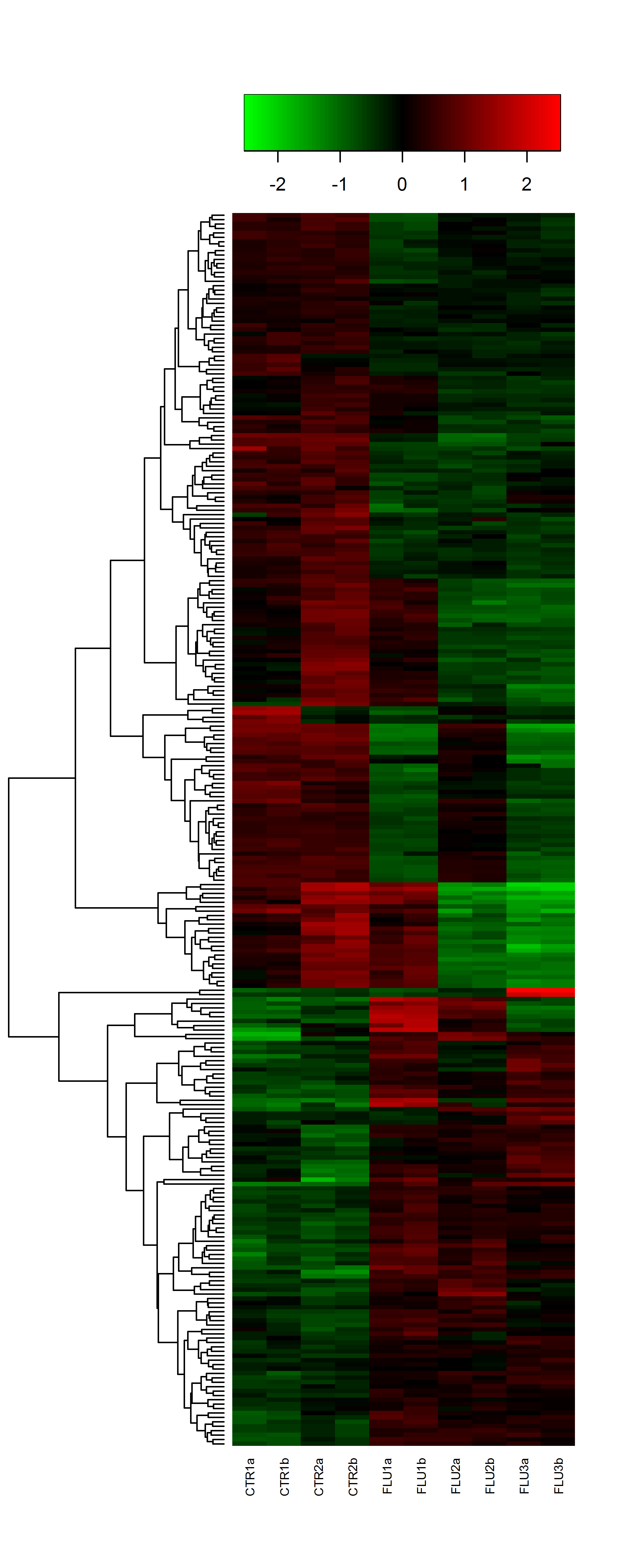

Supplement: Supplementary file 2 — Heatmap illustrating the expression profile of all 280 differentially expressed genes (DEGs; P-adjusted < 0.05 and log2 fold change ≥1.0) in the ovaries of porcine piglets treated with flutamide. The red blocks represent up-regulated genes, and the green blocks represent down-regulated genes. The color scale represents the expression level, where the most bright green stands for − 2.0 log2 fold change and the most bright red stands for 2.0 log2 fold change. (PNG 68 kb) [file 40104_2019_340_MOESM2_ESM.png]

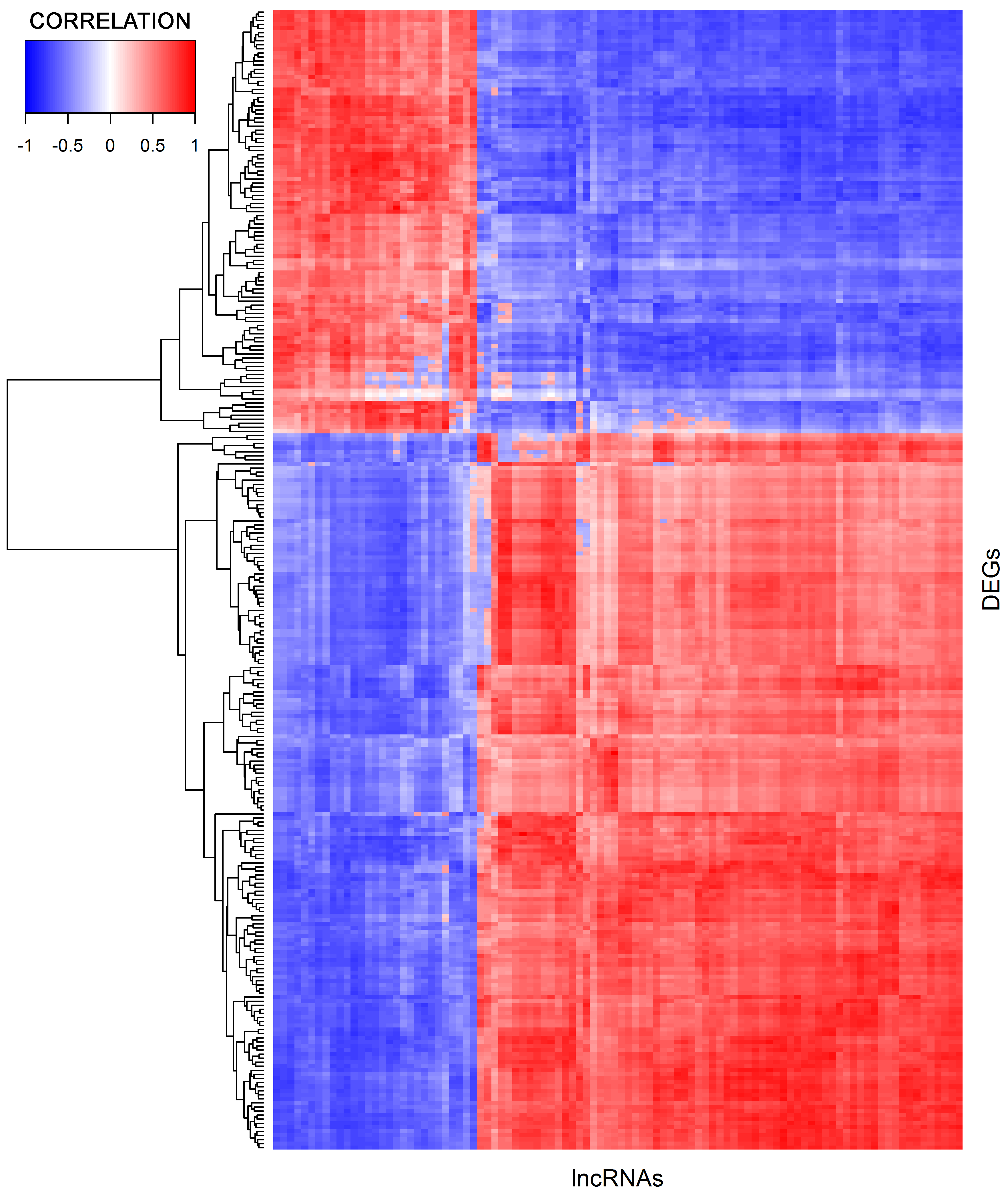

Supplement: Supplementary file 4 — Heatmap illustrating correlations found between 280 differentially expressed genes (DEGs; P-adjusted < 0.05 and log2 fold change ≥1.0) and 98 differentially expressed long non-coding RNAs (DELs; P-adjusted < 0.05 and log2 fold change ≥1.0) in the ovaries of porcine piglets treated with flutamide. The red color represents a positive correlation and the blue color represents a negative correlation. (TIF 1011 kb) [file 40104_2019_340_MOESM4_ESM.tif]

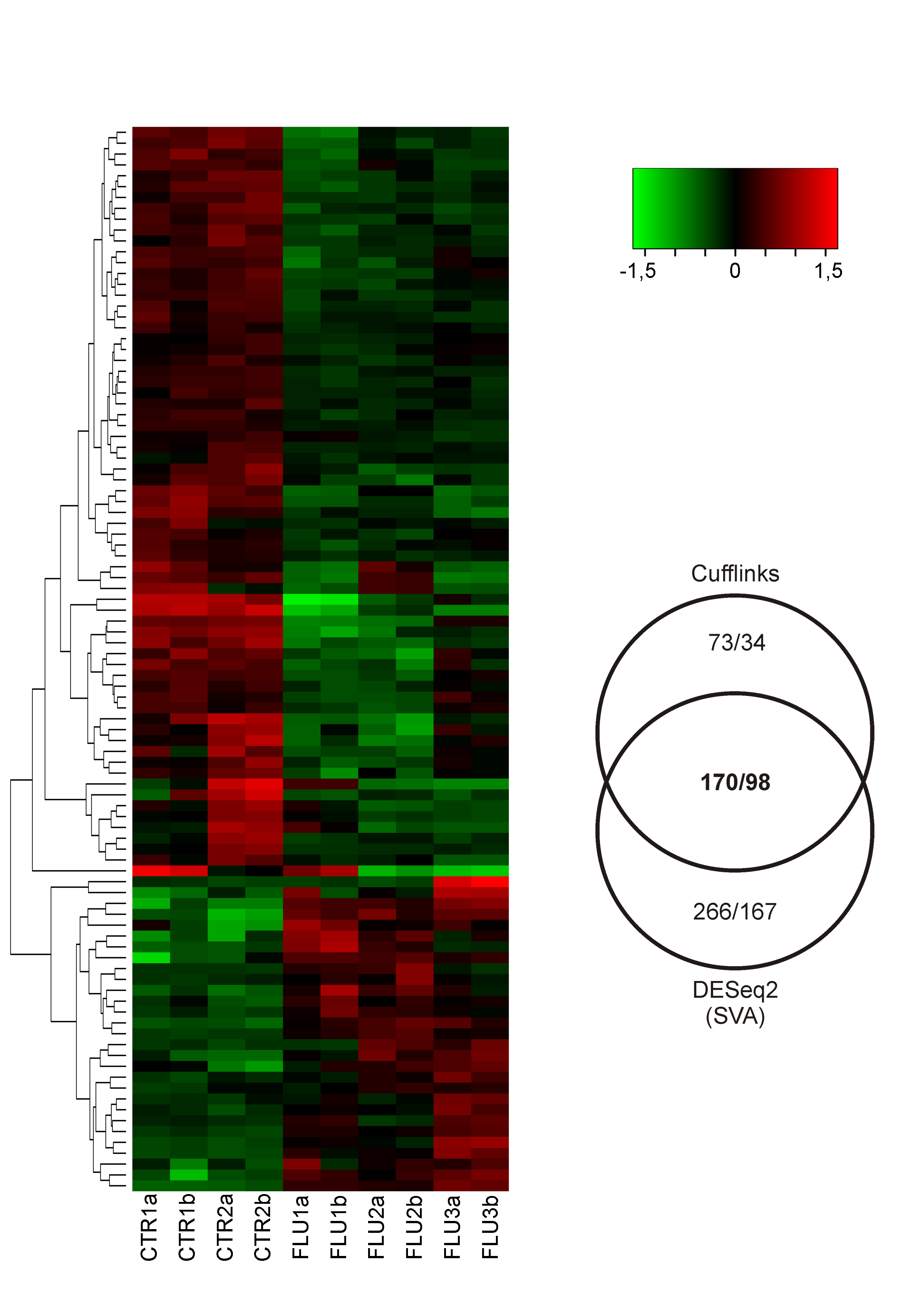

Supplement: Supplementary file 5 — Differentially expressed lncRNAs (DELs; P-adjusted < 0.05 and log2 fold change ≥1.0) in the ovaries of porcine neonates treated with flutamide. The left panel shows a heatmap illustrating the expression profile of all DELs: the red blocks represent up-regulated DELs, and the green blocks represent down-regulated DELs; the color scale of the heatmap represents the expression level, where the most bright green stands for − 2.0 log2 fold change and the most bright red stands for 2.0 log2 fold change. The right panel presents the number of DETs/DELs obtained by employing two statistical methods, i.e., Cufflinks and DESeq combined with SVA batch normalization effect. (TIF 461 kb) [file 40104_2019_340_MOESM5_ESM.tif]
